# A Practical Treatise on Nervous Exhaustion (Neurasthenia)

**Published:** 1889-04

**Authors:** 


A PRACTICAL TREATISE ON NERVOUS EXHAUSTION (NEURASTHENIA) its Symptoms, Nature, Sequences and Treatment, by Geo. M. Beard, A. M., M. D., New York, edited with notes and additions by A. D. Rockwell, A. M., M. D., New York, Publisher by E. B. Treat, N. Y., p. p. 254, prize $2 75.

The first 175 pages are taken up with the symptoms, nature, diagnosis, prognosis and sequences, principly of the symptoms. The rest of the book is devoted to the treatment and hygiene. The word pathology is not used often. It is a book of symptoms, but if one will read it through he will feel that he has been somewhat benifited, yet the idea will prevail that Beard thought a great deal of Beard and that Rockwell is following in his footsteps. B.

